# Novel Triamcinolone Acetonide-Loaded Liposomes Topical Formulation for the Treatment of Cystoid Macular Edema After Cataract Surgery: A Pilot Study

**DOI:** 10.1089/jop.2018.0101

**Published:** 2019-03-14

**Authors:** Alejandro Gonzalez-De la Rosa, Jose Navarro-Partida, Juan Carlos Altamirano-Vallejo, Ada Gabriela Hernandez-Gamez, Jesus Javier Garcia-Bañuelos, Juan Armendariz-Borunda, Arturo Santos

**Affiliations:** ^1^ Tecnologico de Monterrey, Escuela de Medicina y Ciencias de la Salud, Zapopan, México.; ^2^ Centro de Retina Medica y Quirúrgica, S.C., Centro Medico Puerta de Hierro. Zapopan, Jalisco, México.; ^3^ Facultad de Medicina, Universidad Autónoma de San Luis Potosí, San Luis Potosí, México.; ^4^ Instituto de Biología Molecular y Terapia Génica, Centro Universitario de Ciencias de la Salud, Universidad de Guadalajara, Guadalajara, México.

**Keywords:** drug delivery, liposomes, macular edema, topical liposomes formulation, pseudophakic cystoid macular edema

## Abstract

***Purpose:*** To report tolerability, safety, and efficacy of a topical triamcinolone acetonide-loaded liposomes formulation (TA-LF) in targeting the macular area in patients with refractory pseudophakic cystoid macular edema (PCME).

***Methods:*** For tolerability, safety and efficacy evaluation, 12 eyes of 12 patients with refractory PCME were exposed to one drop of TA-LF (TA at 0.2%) every 2 h for 90 days or until best-corrected visual acuity (BCVA) was achieved. Intraocular pressure (IOP), slit lamp examination, and central foveal thickness (CFT) were analyzed at every visit.

***Results:*** Patients with refractory PCME under TA-LF therapy showed a significant improvement in BVCA and CFT without significant IOP modification (*P* = 0.94). On average CFT decreased to 206.75 ± 135.72 μm and BCVA improved to 20.08 ± 10.35 letters (*P* < 0.0005). BCVA was achieved at 10.58 ± 6.70 weeks (range 2–18). TA-LF was well tolerated in all cases. Neither ocular surface abnormalities nor adverse events were recorded.

***Conclusion:*** TA-LF was well tolerated and improved BCVA and CFT on patients with refractory PCME.

## Introduction

Pseudophakic cystoid macular edema (PCME), also called Irvine-Gass syndrome, is the most common cause of decreased central visual acuity (CVA) following a cataract surgery. The incidence of clinical PCME, defined by symptomatic vision loss, is reported at 1.17%–4.04%.^1^ However, the incidence of PCME diagnosed by optical coherence tomography (OCT) can reach 10.9%.^2^ Onset of clinically significant PCME is generally 4–12 weeks after surgery and reaches its peak at 4–6 weeks postoperatively. Patients typically complain of impaired vision after an initial postoperative period of improvement.^3^

Many risk factors have been associated with PCME occurrence, including systemic diseases such as diabetes mellitus,^1,4^ YAG capsulotomy, or preexisting conditions such as uveitis.^1,5^ PCME pathogenesis is unclear, but involves the production of prostaglandins (PGs), cytokines, and other factors released during a surgical trauma that disrupt the blood–retina barrier and macular traction from prolapsed or incarcerated vitreous.^3^

Available therapeutic interventions, both for prophylaxis and for the treatment of PCME, are based on the postulated pathogenesis of the condition. PCME management includes pharmacological and non-pharmacological strategies. Although the best therapeutic options for treating this disorder have not been established, corticosteroids and topical nonsteroidal anti-inflammatory drugs (NSAIDs), either as monotherapy or in combined therapy, are widely used as first-line treatment for acute PCME (within 6 months postoperatively).^6^

Nevertheless, to date, there are no evidence-based recommendations as to which patients should be treated, neither about the optimum postoperative timing of treatment initiation.^7^ PCME is called refractory when topical treatment is ineffective. Intravitreal corticosteroids are efficient for this condition. Pars plana vitrectomy is indicated for chronic PCME (> 6 months), refractory PCME and PCME associated with vitreomacular traction.^6^

The presumed therapeutic activity of corticosteroids in PCME is related to the blockage of leukotriene and PG synthesis by inhibiting phospholipase A2 in the arachidonic acid cascade, as well as the reduction of macrophage and neutrophil migration, and capillary permeability and vasodilation. The therapeutic activity of NSAIDs in PCME is related to the inhibition of cyclooxygenase enzymes (COX-1 and COX-2). Both enzymes catalyze the biosynthesis of eicosanoids from arachidonic acid to produce PGs and thromboxanes that cause vasodilatation and disruption of the blood–ocular barrier.^6^

It is important to emphasize that therapy combining topical steroids and NSAIDs is presumably superior to either treatment alone for PCME. For example, the combination of ketorolac and prednisolone has resulted in an improvement of 3.8 Snellen lines and a quicker response, compared with 1.6 Snellen lines with ketorolac and 1.1 lines with prednisolone in patients with acute PCME.^8^

In contrast, intravitreal corticosteroids without NSAIDs have been used successfully to treat PCME. Different reports using intravitreal triamcinolone acetonide (IVTA) have shown high efficacy against refractory PCME, significant improvement in visual acuity, and significant reduction in macular thickness.^9–12^ Different biological and therapeutic activities have been related to IVTA, such as inhibiting the breakdown of the blood–retinal barrier in diabetic rat retinas through the regulation of vascular endothelial growth factor-A (VEGF-A) and its receptors^13^ and preventing choroidal neovascularization in a laser-treated rat model.^14^ However, IVTA is associated with increased intraocular pressure (IOP) that requires, in most cases, topical IOP-lowering drugs.^9,12^

Although intravitreal injection of triamcinolone acetonide (TA) is a well-described and effective route to release this corticosteroid into the vitreous cavity, this procedure is not without severe potential complications, such as endophthalmitis, lens injury, and retinal detachment.^15–17^ Additionally, clinical studies have related the use of intravitreal TA with IOP increase, cataract formation or progression, and non-infectious endophthalmitis.^18–20^

To diminish ocular hazards related to intravitreal injections of TA but retaining the benefits of TA in refractory PCME, it is necessary to develop alternative strategies for drug delivery. Recently, a topical triamcinolone acetonide-loaded liposomes formulation (TA-LF) was used to deliver TA into vitreous and retina of rabbits.^21^ Although the therapeutic activity of TA against vitreoretinal diseases, including refractory PCME, is well known,^9–12,22–24^ the biological and therapeutic activity of TA-LF has not been confirmed. Therefore, the aim of this study was to report the evaluation of tolerability and safety of TA-LF for ophthalmic use and to explore its therapeutic efficacy in targeting the macular area in patients with refractory PCME.

## Methods

### Study design

To evaluate tolerability, safety, and efficacy of a novel topical TA-LF in the treatment of macular edema, a single-center prospective pilot study was conducted on patients diagnosed with refractory PCME at a private-based retina specialty center in Guadalajara, Mexico (Centro de Retina Medica y Quirurgica S.C.). An external review board approval and Ministry of Health approval was obtained before enrollment of patients (COFEPRIS 173300410A0035/2017). It is important to emphasize that this study adhered to the tenets of the Declaration of Helsinki.

### Patients

Patients with refractory PCME were enrolled 60–90 days after uncomplicated phacoemulsification and capsular bag lens implantation. Refractory PCME was defined as central foveal thickness (CFT) ≥300 μm, measured by optical coherence tomography (Cirrus OCT Carl Zeiss, Meditec, Dublin, CA), and a registered increase in >8 μm or changes of ±7.9 μm in CFT, after 4 weeks of topical NSAID therapy (nepafenac 0.1%, 3 times a day).^25,26^ Fluorescein angiography (FA) was performed at baseline to confirm the angiographic pattern of macular edema in all cases. After full explanation of the nature and possible consequences of the study, written informed consent was obtained from the participants.

Demographic and baseline clinical exams were collected for enrolled patients 1–3 days before the establishment of TA-LF therapy. TA-LF administration started 48 hrs after the last instillation of topical nepafenac to permit its clearance from ocular tissues.^27^ Victrectomized patients were included when the indication for surgery was vitreous floaters. The regimen of TA-LF therapy was 1 drop every 2 h (6 times a day) for a period of at least 12 weeks or until final best-corrected visual acuity (BCVA) was achieved (vision improvement arrested for 4 weeks with continuous treatment). Final TA concentration in the used formulation (TA-LF) was 2 mg/mL (0.2%). This dose was based on the preclinical data from a pharmacokinetic study on rabbits.^21^

Exclusion criteria were Snellen visual acuity >20/40 (>70 letters in the ETDRS chart), FA or retinal OCT not consistent with CME, use of topical steroid 1 month before the study, placement of steroid ocular implant 12 months before study enrollment, use of intraocular corticosteroids or anti-angiogenic drugs 3 months before the study, vitrectomy for floaters 1 year before the study, ocular disease preventing an adequate examination of the fundus, any ocular disease that could be responsible for decreased visual acuity (diabetic retinopathy, vascular occlusion, macular degeneration), ocular hypertension, glaucoma, and unstable systemic diseases, including systemic hypertension, diabetes mellitus, and previous eye disease resulting in a medical history of CME. Patients with previous cerebrovascular accident or myocardial infarction were also excluded.

### Efficacy assessment

To evaluate the therapeutic efficiency of TA-LF in refractory PCME, a follow-up with CFT and visual acuity was performed. The BCVA using ETDRS chart at 4 m and the average CFT by OCT were measured at baseline and during every visit. Study visits were scheduled every week during the first month and every month during the rest of the follow-up period (20 weeks). Additionally, IOP, slit lamp anterior, and posterior segment evaluation were recorded at each visit with the purpose of identifying ocular adverse events (AEs).

### Safety and tolerability assessment

Tolerability was assessed through the collection and summary of ocular and non-ocular AEs, serious AEs (SAEs), ocular assessments and vital signs, whether volunteered by the enrolled patients, discovered by study site personnel during questioning, or other means. Subjects were withdrawn if they presented any evidence of poor tolerability or any AE, such as corneal ulcers, corneal opacities, epithelial defects, anterior chamber inflammation (cell/flare), and conjunctival and/or epiescleral infection related to the use of this topical formulation. AEs were assigned standard codes for the event based upon the MedDRA Coding dictionary, version 18.1.

### Rescue treatment

Rescue treatment with intravitreal injection of 4 mg of preservative-free TA was considered when patients showed the following characteristics: worsening of BCVA >15 letters or increase in CFT by OCT (>70 μm compared with baseline), lack of BCVA changes after 4 weeks of TA-LF therapy, or changes of ≤7.9 μm in CFT after 4 weeks of TA-LF administration.

IOP-lowering drugs were considered when registered IOP was ≥22 mmHg or >4 mmHg when compared with contralateral eye.

### Preparation of liposomal formulation

OPKO Health, Inc. (Guadalajara, Jalisco, Mexico) provided a TA-LF. Preparation of TA-LF was carried out as previously described.^21^ Briefly, self-forming, thermodynamically stable TA-LFs (QuSomes^®^) were generated spontaneously upon adding polyethylene glycol glyceryl dimyristate (PEG-12) to an aqueous solution containing TA. Composition of TA-LF is described in Table 1. Final TA concentration in the resultant dispersion was 2 mg/mL (0.2%).

**Table 1.** Composition of Triamcinolone Acetonide-Loaded Liposomes Formulation

*Reagent*	*Volume*
Triamcinolone acetonide	2.0 mg
Kolliphor HS 15	50 mg
PEG-12 glyceryl dimyristate	100 mg
Ethyl alcohol	14 μL
Citric acid anhydrous	0.8 mg
Sodium citrate dihydrate	4.675 mg
Benzalkonium chloride	0.1 mg
Grade 2 purified water	Q.S.1.0 mL

### Statistical analysis

Data were analyzed using the SPSS 22.0 software (IBM SPSS Statistics for Macintosh, version 22.0, IBM Corp, Armonk, NY). Quantitative variables were described using mean and standard deviation. Qualitative variables were described using frequencies and percentages. We performed a Wilcoxon signed-rank test and Mann–Whitney U-test for the analysis of age, BVCA, CFT, IOP, and weeks to BCVA in dependent and independent samples, respectively. For the analysis of gender and study eye, a Fisher exact test was performed. Significance was defined as a *P*-value <0.05.

## Results

Twelve eyes of 12 patients with refractory PCME were analyzed. These subjects were instructed to apply one drop of TA-LF every 2 h in the study eye, while awake (6 times a day), for at least 12 weeks or until they reach their final BCVA (final BCVA was considered when vision improvement was arrested for 4 weeks with continuous treatment). The male–female ratio of this group was 5:7, and the mean age was 68.08 ± 12.16 years. Five of the 12 study eyes were right and 7 were left. Patient demographics and characteristics are summarized in Table 2.

**Table 2.** Demographics and Clinical Characteristics of Patients with Pseudophakic Cystoid Macular Edema Treated with Triamcinolone Acetonide-Loaded Liposomes Formulation

*Patient*	*Gender*	*Age (years)*	*Study eye*	*Baseline*				*20 weeks of follow-up*							
				*CFT (μm)*	*BCVA (ETDRS letters)*	*IOP (mmHg)*	*Vitrectomy*	*CFT (μm)*	*CFT change (μm)*	*BCVA (ETDRS letters)*	*BCVA change (ETDRS letters)*	*Weeks to BCVA*	*IOP*^a^*(mmHg)*	*IOP change (mmHg)*	*AEs*
1	F	63	OD	545	52	17	Yes	267	−278	59	7	2	12	5	No
2	F	76	OD	580	46	15	Yes	319	−261	75	29	7	11	4	No
3	M	59	OS	461	43	12	Yes	322	−139	58	15	17	11	1	No
4	F	58	OS	369	49	15	Yes	360	−9	78	29	2	17	−2	No
5	F	62	OS	605	53	13	No	248	−357	73	20	13	14	−1	No
6	F	74	OD	487	50	12	Yes	268	−11	79	29	2	11	1	No
7	F	78	OS	600	54	14	No	250	−350	60	6	17	16	−2	No
8	M	59	OS	415	47	14	No	385	−30	75	28	18	13	1	No
9	M	64	OS	354	53	17	No	262	−92	70	17	18	13	4	No
10	F	66	OD	666	52	11	No	253	−413	65	13	13	18	−7	No
11	M	60	OD	596	50	14	No	336	−260	60	10	11	18	−4	No
12	M	64	OS	360	45	12	No	287	−73	83	38	3	13	−1	No
	M: 5 (41.66%)	68.08 ± 12.16	OD: 5 (41.66%)	503.17 ± 110	49.5 ± 3.55	13.83 ± 1.95	Yes: 5 (41.66%)	296 ± 46.70^b^	−206.75 ± 135.72	69.58 ± 8.84^b^	20.08 ± 10.35	10.25 ± 6.70	13.92 ± 2.68^c^	−0.08 ± 3.50	Yes: 0 (0.00%)
	F: 7 (58.33%)		OS: 7 (58.33%)				No: 7 (58.33%)								No: 12 (100%)

^a^ IOP at 20 weeks of follow-up.

^b^ Statistically significant differences from baseline values (*P* < 0.0005).

^c^ No statistically significant differences from baseline values (*P* > 0.05).

F, female; M, male; OD, right eye; OS, left eye; CFT, central foveal thickness; BCVA, best-corrected visual acuity; ETDRS, Early Treatment Diabetic Retinopathy Study; IOP, intraocular pressure; TA-LF, triamcinolone acetonide-loaded liposomes formulation; AEs, adverse events.

Related to tolerability and safety outcomes, we observed that the TA-loaded LF was well tolerated during the study period. Neither ocular (increased intraocular pressure) nor systemic AEs were reported. None of the 12 patients showed significant changes in IOP (13.83 ± 1.95 vs. 13.92 ± 2.68; *P* = 0.94). After using the study formulation, none of the patients required IOP-lowering drugs. None of the patients showed signs of irritation or surface problems due to the study formulation application until the end of the study.

**FIG. 1.**
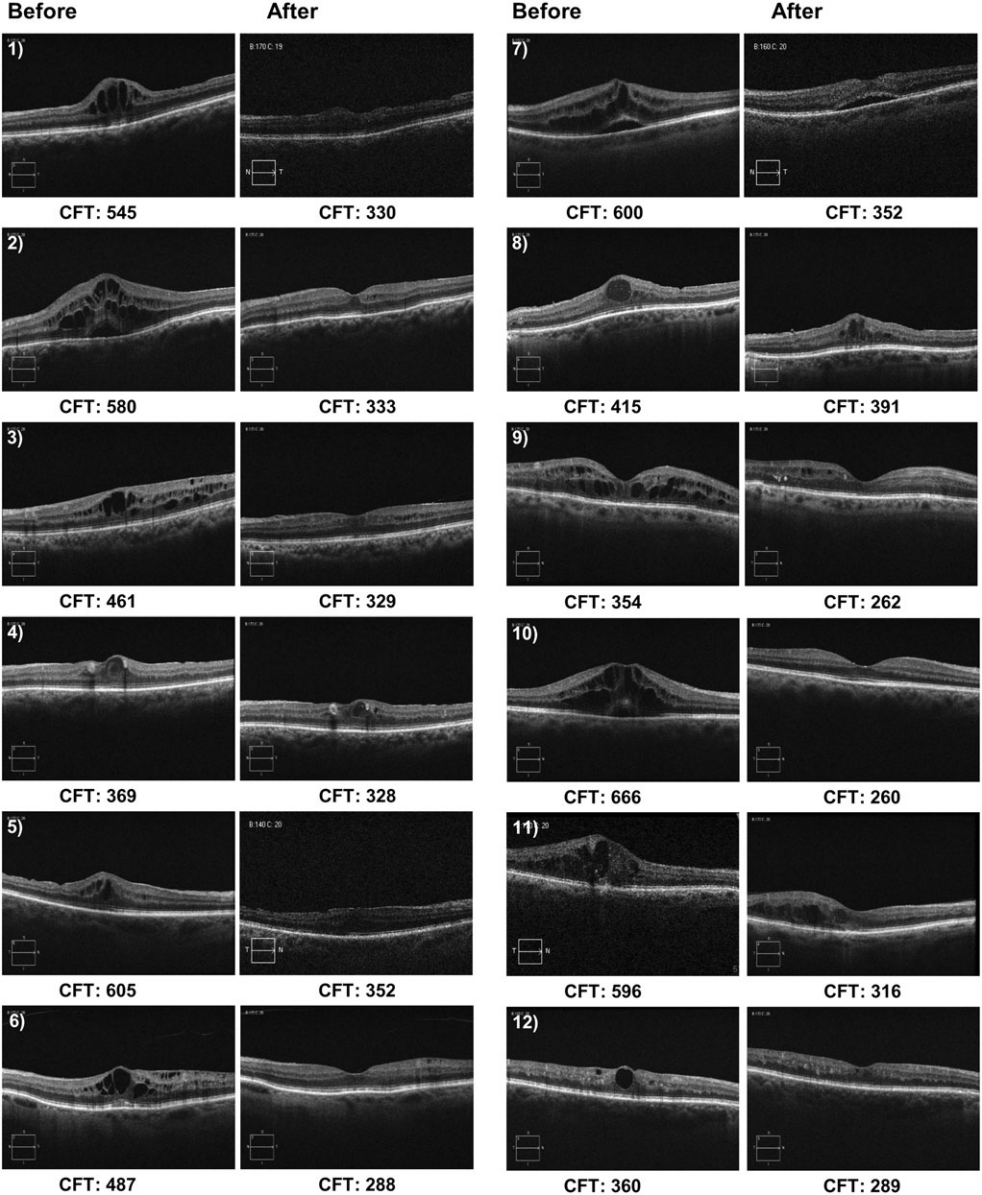
OCT images before and after TA-LF therapy. The figure is composed of representative retinal OCT images of all cases of PCME before and after topical TA-LF therapy. Measurement of CFT is presented in μm. CFT, central foveal thickness; PCME, pseudophakic cystoid macular edema; OCT, optical coherence tomography; TA-LF, triamcinolone acetonide-loaded liposomes formulation.

In contrast, we found that TA-LF showed a satisfactory therapeutic activity. All 12 patients under TA-LF therapy showed a decrease in CFT documented by OCT at 20 weeks of follow-up (503.17 ± 110 vs. 296 ± 46.70 μm; *P* < 0.0005). The average change in CFT was a decrease of −206.75 ± 135.72 μm. By OCT criteria, none of the patients needed rescue treatment (increase of >70 μm compared with baseline). Representative OCT images of the 12 patients are shown in Fig. 1.

**FIG. 2.**
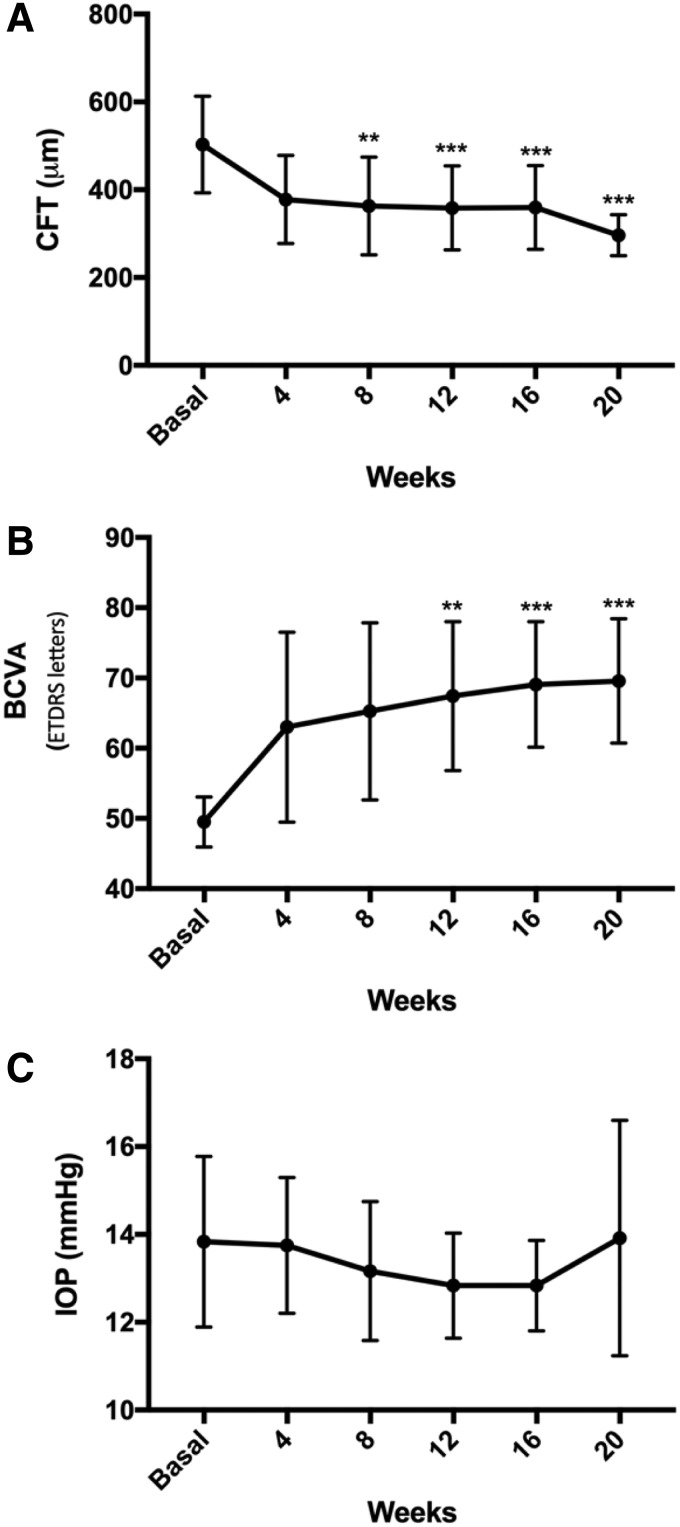
Variations in CFT, BCVA, and IOP during follow-up TA-LF therapy in patients with refractory PCME. **(A)** A significant reduction in CFT began at week 8. **(B)** A significant increase in BCVA was registered at 12 weeks. **(C)** Nonsignificant variations in IOP were recorded during the follow-up. **Statistically significant differences from baseline values (*P* < 0.001), ***statistically significant differences from baseline values (*P* < 0.0001). BCVA, best-corrected visual acuity; ETDRS, Early Treatment Diabetic Retinopathy Study; IOP, intraocular pressure.

Additionally, all patients showed BCVA improvement. The shortest time to reach BCVA was 2 weeks, while the longest response time recorded was 18 weeks (average of 10.25 ± 6.70 weeks). The mean change in BCVA was 20.08 ± 10.35 letters (49.50 ± 3.55 vs. 69.58 ± 8.84 ETDRS letters; *P* < 0.0005). Clinical characteristics of refractory PCME patients after TA-LF treatment are summarized in Table 2. Variations in CFT, BCVA, and IOP during follow-up TA-LF therapy are presented in Fig. 2.

Finally, because pharmacokinetic studies have suggested that the half-live of drugs is shortened in vitrectomized eyes due to a faster drug clearance compared with nonvitrectomized eyes, we decided to perform a stratified analysis.^28–30^ We noticed that vitrectomized eyes tended to improve in a shorter period of time (7.20 ± 7.25 weeks for vitrectomized vs. 12.43 ± 5.83 weeks for nonvitrectomized; *P* = 0.1250); this condition was also associated with a lower increment in IOP (12.40 ± 2.61 vs. 15.00 ± 2.31 mmHg for vitrectomized and nonvitrectomized patients, respectively; *P* = 0.045).

As expected, therapeutic activity was similar for both vitrectomized and nonvitrectomized patients. Reduction in CFT (307.20 ± 39.68 vs. 288.71 ± 52.68; *P* = 0.99) and BCVA improvement (69.80 ± 10.43 vs. 69.43 ± 8.42; *P* = 0.625) was similar for both groups. Comparisons of clinical characteristics of patients vitrectomized and nonvitrectomized at baseline and after TA-LF treatment are summarized in Table 3.

**Table 3.** Comparison of Clinical Characteristics of Vitrectomized and Nonvitrectomized Patients Treated with Triamcinolone Acetonide-Loaded Liposomes Formulation

	*Vitrectomized*	*Nonvitrectomized*	P *value*
Baseline			
Gender			
M (*n*)	1.00	4.00	0.2922
F (*n*)	4.00	3.00	
Study Eye			
OD (*n*)	3.00	2.00	0.5581
OS (*n*)	2.00	5.00	
Age (years)	66.00 ± 8.46	64.71 ± 6.34	0.7854
CFT (μm)	488.40 ± 81.55	513.71 ± 132.05	0.6389
BCVA (ETDRS letters)	48.00 ± 3.54	50.57 ± 3.41	0.1831
IOP (mmHg)	14.20 ± 2.17	13.57 ± 1.90	0.5164
Therapy results			
CFT at 20 weeks of follow-up (μm)	307.20 ± 39.68	288.71 ± 52.68	0.9990
BCVA (ETDRS letters)	69.80 ± 10.43	69.43 ± 8.42	0.6250
Weeks to BCVA	7.20 ± 7.25	12.43 ± 5.83	0.1250
^a^IOP	12.40 ± 2.61	15.00 ± 2.31	0.0450

^a^ IOP at 20 weeks of follow-up.

## Discussion

The introduction of phacoemulsification significantly decreased the incidence of PCME. However, it remains the most frequent postoperative complication leading to impaired vision. Although mostly self-limited,^31^ persisting and refractory cases represent a therapeutic challenge and are associated with substantial costs for health care systems.^4^

A wide range of pharmacologic agents have been used for PCME treatment, including steroids (prednisolone, TA, and dexamethasone),^8,11,32^ NSAIDs,^33–35^ carbonic anhydrase inhibitors,^36^ monoclonal antibodies against VEGF,^37,38^ and tumor necrosis factor-alpha.^39^ Studies testing the efficacy of these interventions have yielded inconclusive results. Thus, there is no widely accepted treatment. However, all therapeutic agents aim to decrease macular edema and thereby improving visual acuity.

Corticosteroids are potent antiangiogenic and anti-inflammatory molecules that play a major role in the management of different vitreoretinal diseases due to its ability to regulate the expression of key genes such as VEGF and interleukin-6.^40,41^ However, topical, oral, or parenteral corticosteroids barely get into the posterior ocular segment due to both ocular and blood–retinal barriers.

However, intravitreal injections of steroids reach suitable intraocular concentrations avoiding the ocular barriers.^42^ Intravitreal TA has been found to be suitable for refractory PCME treatment.^9,12^ Nevertheless, to diminish ocular hazards related to intravitreal injections of TA and to preserve the benefits of using them in refractory PCME, it is necessary to develop alternative strategies for drug delivery.

Liposomes (LPs)-based eye drops have been proposed as a new system of drug delivery into the posterior segment of the eye, and they have the potential to deliver drugs in therapeutic concentrations to the vitreous cavity and retina.^21^ LPs are particles composed of an aqueous core and delimited by a membrane-like lipid bilayer that works as carriers for water-soluble, lipid-soluble, and amphiphilic drugs.^43–46^ Because of their resemblance to biomembranes, LPs are non-toxic, low antigenic, easily metabolized, and biodegradable.^47^ They have been employed to improve drug transport and bioavailability in ocular tissues.^48,49^

A recent study of a novel topical TA-LF reported a suitable route for ophthalmic use to release TA in a controlled manner. Authors have reported that TA-LF is capable of releasing TA efficiently in the vitreous and retina. However, its safety and tolerability in humans, as well as its biological and therapeutic activity, has not been confirmed.^21^ In this report, we found that TA-LF is suitable for ophthalmic use in human with satisfactory tolerability profile. No evidence of systemic or ocular AEs, such as intraocular hypertension or visual acuity loss, was recorded in treated patients.

Interestingly, TA-LF improved BCVA and reduced CFT in patients with refractory PCME, proving its therapeutic activity. BCVA and CFT of refractory PCME patients, at no point, were worse than baseline in the treated eye. Moreover, the treated eyes showed BCVA improvement of 4–35 letters, and CFT decreased an average of 184 ± 113.82 μm. It is important to highlight that in some cases the correlation between BCVA and CFT was weak. However, it is well known that the changes in BCVA and CFT are weakly correlated regardless of the underlying disease etiology.^50–53^

In the past, topical corticosteroids have been tested as mono and adjuvant therapy for acute PCME. For example, combined topical therapy for acute PCME consisting of prednisolone acetate 1.0% and ketorolac tromethamine 0.5% has been shown to be quicker and more effective than either treatment alone. In fact, in patients who improved 2 lines or more, such improvement occurred in an average time of 2.75 months after initiating prednisolone therapy, 1.43 months with ketorolac instillation, and 1.33 months with combined therapy.^8^

In contrast, in a recent study on the prevention of PCME after cataract surgery in nondiabetics that included 914 patients, the combination of topical bromfenac 0.09% and dexamethasone 0.1% was related to lower incidence of clinically significant macular edema 12 weeks postoperatively (1.5%). This finding supports the hypothesis that NSAIDs and corticosteroids are more effective in combination than either formulation alone.^54^ However, the therapeutic action of topical steroids and NSAIDs alone or in combination seemed to be lesser in chronic or refractory PCME.^55^

Injection of periocular corticosteroids is a viable option for PCME that is resistant to topical medication. Retrobulbar and subtenon injections of steroids are efficient against refractory PCME, but they are frequently associated with IOP rise.^56^ Also, IVTA has been shown to be effective against refractory PCME. A significant improvement in visual acuity and retinal thickness has been documented with this strategy.^9–12^ However, IVTA has caused IOP to rise in a third of treated patients.^9,12^

Dexamethasone as a biocompatible intravitreal implant (Ozurdex^©^, Allergan, Irvine, CA) has been used successfully in treating chronic and refractory PCME and is less associated with IOP rise.^32,57,58^ This implant slowly released 0.7 mg over a period of up to 6 months. Besides, the Ozurdex implant in comparison with IVTA has demonstrated equal efficacy in diabetic patients with PCME.^59^

Although dexamethasone intravitreal implant is a valid strategy in the treatment of chronic and refractory PCME, presumably the costs and risks associated with its insertion, in comparison with a topical NSAID or topical corticosteroid formulation, limit the wider use of this therapeutic option in PCME. It is important to consider that the placement of dexamethasone intravitreal implant requires highly qualified and specialized professionals as well as an operating room, whereas topical formulation does not involve specialized infrastructure and it could be self-administered.

In this study, we found that patients with refractory PCME under topical therapy with TA-LF improved their BCVA and reduced their CFT in a significant manner. Moreover, they reached their BCVA within 2 (range 2–18) weeks (16.66%) and improved 22.33 ± 4.32 letters without significant increase in IOP during the follow-up period. It is possible that this delivery method of smaller amounts of drug (as it occurs with the dexamethasone intravitreal implant) could have prevented IOP rise.

Therefore, TA-LF could be not only an effective alternative but also a safer strategy than injected steroids for the treatment of refractory PCME, because topical formulations do not have the potential hazards associated with intravitreal injections such as endophthalmitis, lens injury, and retinal detachment.^15–17^ However, complementary studies need to be performed to confirm this statement.

Additionally, an important issue to discuss is the finding of differences in efficacy between vitrectomized and nonvitrectomized eyes. Pharmacokinetic studies have suggested that the half-live of drugs is shortened in vitrectomized eyes due to a more rapid drug clearance compared with nonvitrectomized eyes; consequently, repeated doses of drugs are necessary.^28–30^ For instance, studies have reported that the concentration of IVTA decreased faster in the vitrectomized eye than in the nonvitrectomized eye,^30^ and that vitrectomized eyes needed more dexamethasone implants compared with nonvitrectomized eyes for the treatment of CME of various etiologies (1.35 vs. 3.2 dexamethasone implants).^60^

In this report, we documented similar functional and anatomic improvement in vitrectomized and nonvitrectomized patients; however, vitrectomized patients tended to improve faster than nonvitrectomized patients with lower increments in IOP. This finding is consistent with those reported for other intravitreal drugs. It seems that vitreous acts as a barrier as well as a reservoir for TA release.^21^

Finally, there are several limitations in our study. First, the lack of a control group was a major problem, but due to ethical considerations, neither placebo nor IVTA groups were considered. The second major limitation of our study was the small sample size. Further studies with bigger sample sizes are needed. Finally, longer periods of follow-up should be considered in further studies to evaluate safety and potential AEs.

In conclusion, we presented for the first time a successful topical TA-loaded liposomal formulation for the treatment of patients with CME associated with cataract surgery. The use of a topical ophthalmic formulation was well tolerated and showed an adequate safety profile, with neither ocular AEs nor significant changes in IOP in patients with refractory PCME. Besides, the study formulation was effective in reducing CFT and improving BCVA in patients with refractory PCME. These findings suggest that topical TA-LF could be effective in the treatment of patients with refractory PCME and may be a potential substitute to intravitreal steroids. However, larger clinical trials are needed to evaluate longer-term safety and therapeutic profile of this novel liposomal formulation.
